# Supplementary material for: Prevention of EloR/KhpA heterodimerization by introduction of site-specific amino acid substitutions renders the essential elongasome protein PBP2b redundant in Streptococcus pneumoniae
Source: Sci Rep. 2019 Mar 6;9:3681. doi: 10.1038/s41598-018-38386-6 (PMC6403258; doi:10.1038/s41598-018-38386-6)
Supplement: Supplementary file 1 — Supplemetary file [file 41598_2018_38386_MOESM1_ESM.pdf]

Supplemental Material.

Prevention of EloR/KhpA heterodimerization by introduction of site-specific amino acid substitutions renders the essential elongasome protein PBP2b redundant in *Streptococcus pneumoniae*.

Anja Ruud Winther, Morten Kjos, Gro Anita Stamsås, Leiv Sigve Håvarstein and Daniel Straume.

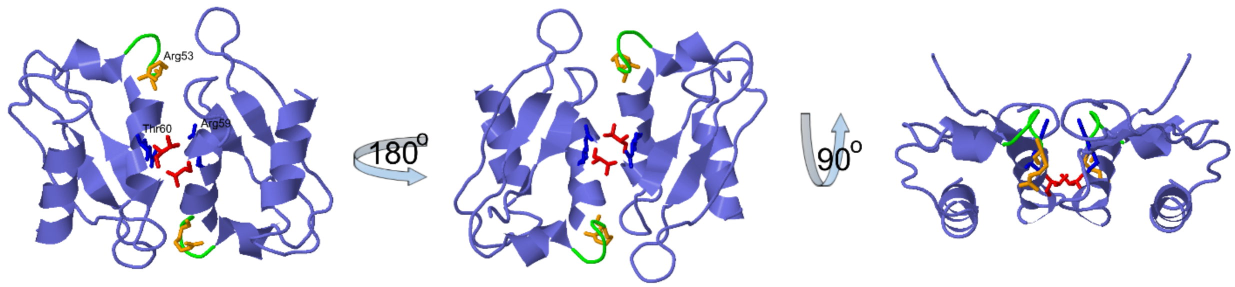

Fig. S1. Model of the KhpA homodimer. The residues R53 (orange), R59 (Blue) and T60 (red) are shown as sticks.

```
KhpA       MDTIENLIIAIVKPLISQPDALTIKIEDTPEFLEYHLNLDQSDVGRVIGRKGRTISAIRT 60
KH-II      --------MAYVQTIIDDMDV-EATLSNDYNRRSINLQIDTNEPGRIIGYHGKVLKALQL 51
                   :* *: :*.: *.   .:.:  :  . :*::* .: **:** :*:.:.*::

KhpA       IVYSVPT-EYKK-VRIVIDEK    79
KH-II      LAQNYLYNRYSRTFYVTI---    69
           :. .    .*.:  . :.*
```

Fig. S2. Alignment of the amino acid sequences of KhpA and KH-II^{EloR} using Clustal Omega[1]. The RNA-binding GXXG loop, and I61 and L239 in KhpA and EloR, respectively, are boxed.

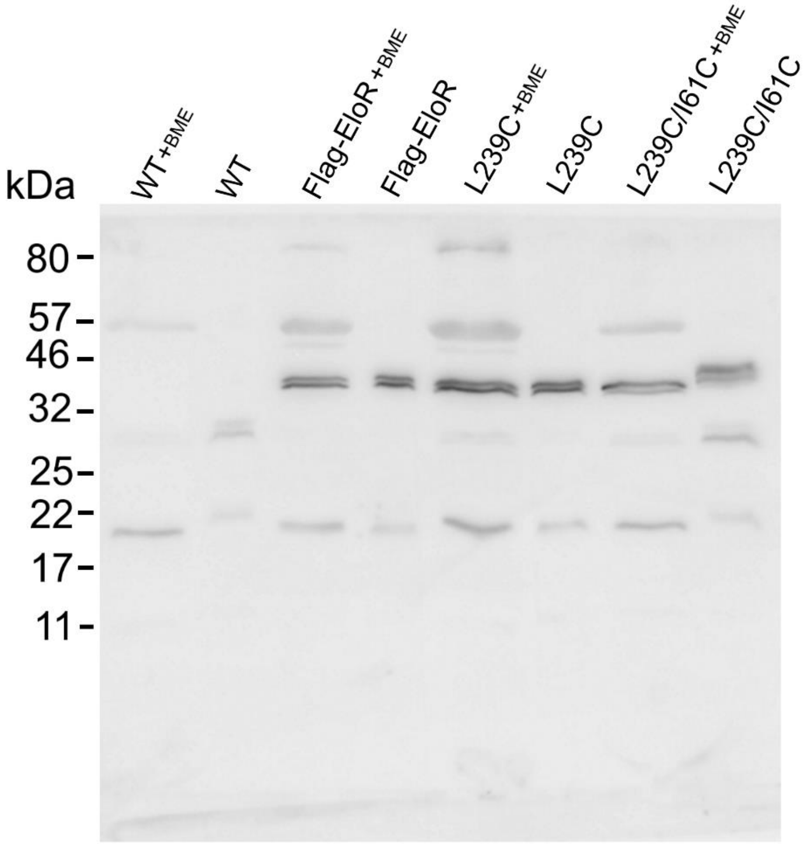

Fig. S3. Full-length image of the immunoblot shown in Fig. 3B.

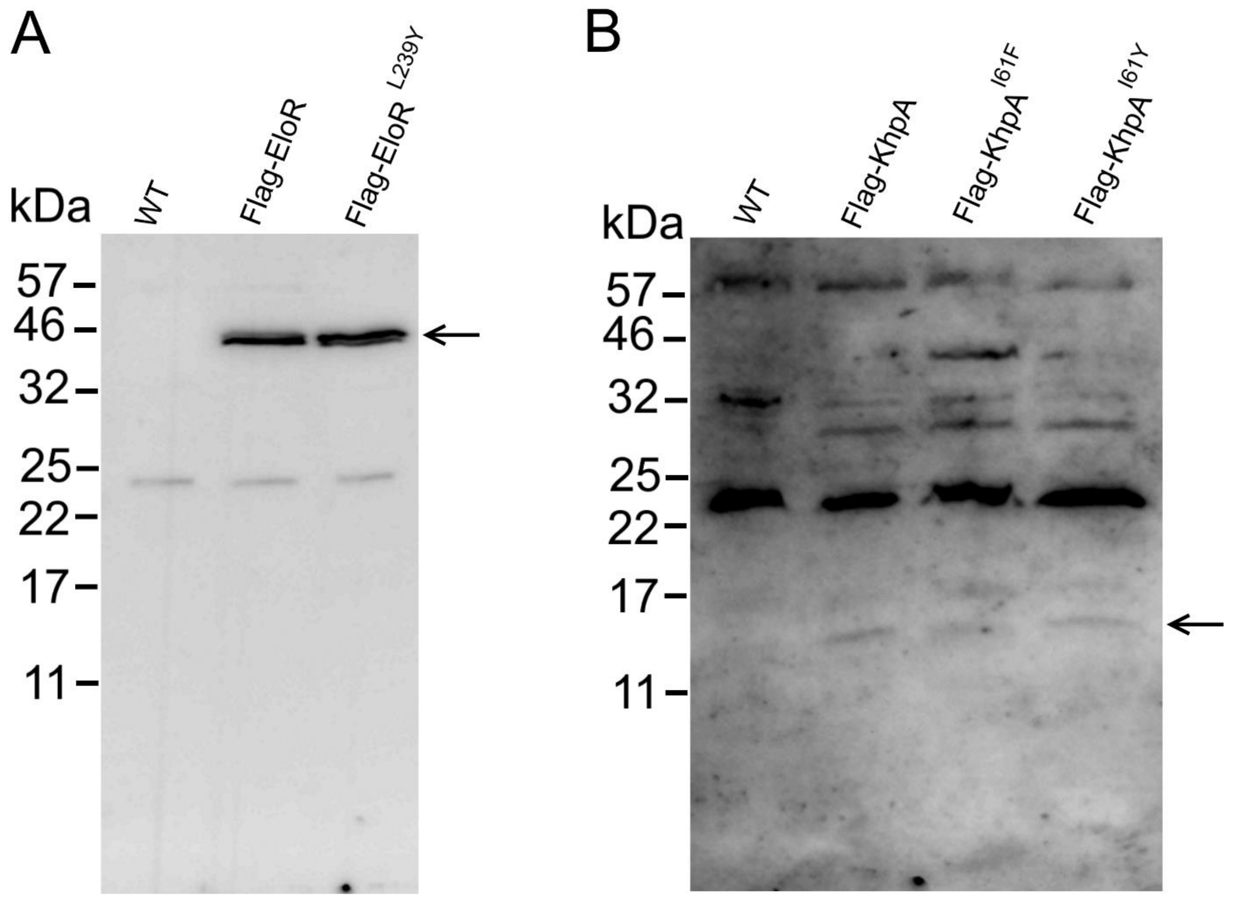

Figure S4. Immunodetection of Flag-EloR and Flag-EloR^{L239Y} is shown in panel A, while detection of Flag-KhpA, Flag-KhpA^{I61F} and Flag-KhpA^{I61Y} is shown in panel B. The arrows indicate Flag-tagged proteins.

Table S1. Suppression of Δ*pbp2b* phenotype when the EloR/KhpA interaction is broken.

Strains[a]	Genotype	Number of normal sized colonies after 18-20 hours of incubation
RH425	R6 derivative, but Sm^R	0
DS420	Δ*comA*, Δ*khpA*; Ery^R, Sm^R	>500
SPH445	Δ*comA*, Δ*eloR*; Ery^R, Sm^R	>500
AW5	Δ*comA, sfgfp-khpA*; Ery^R, Sm^R	0
AW24	Δ*comA, khpAGDDG*; Ery^R, Sm^R	>500
AW212	Δ*comA, khpA*^{I61F}; Ery^R, Sm^R	0
AW275	Δ*comA, khpA*^{I61Y}; Ery^R, Sm^R	>500
AW279	Δ*comA, eloR*^{L239Y}; Ery^R, Sm^R	>500

[a]The strains listed were transformed with a Δ*pbp2b*::janus amplicon as described in materials and methods. The transformations were done at least three times with similar results.

Table S2. Primers used in the present study.

Primer	Sequence (5\` → 3\`)	Reference
**Primers used to create the Δ*khpA*::janus amplicon**		
ds382	ATTTAGGGAACCAGATCTTAAG	This work
ds383	CACATTATCCATTAAAAATCAAACCTGTCAACCTACT TTAAACTTATTTTG	This work
ds384	GTCCAAAAGCATAAGGAAAGGAAGGGCGGGACGGAT GTC	This work
ds385	CAGGACCACACTCGTCAATC	This work
Kan484F	GTTTGATTTTTAATGGATAATGTG	[2]
RpsL41R	CTTTCCTTATGCTTTTGGAC	[2]
**Primers used to create the Δ*pbp2b*::janus amplicon**		
khb129	CGATAAAGAAGAGCATAGGAAG	[3]
khb132	TCCCAATCAATGGTTTCATTGG	[3]
**Primers used to create the Δ*rodA*::janus amplicon**		
ds342	AGAAAGTATTCGCTTTGAGTGC	[4]
ds343	TCCAAAACCTGATCATTTCGATG	[4]
**Primers used to create the Δ*eloR*::janus amplicon**		
ds374	CGAAACCTTGGGATACGCAG	[5]
ds377	CAGCACCCACGTTAAGCAAC	[5]
**Primers used to create the *khpA*^{I61F} amplicon**		
aw130	TTTGTCTACTCTGTCCCAACTGA	This work
aw131	TCAGTTGGGACAGAGTAGACTTTCGTTCTTATCGCAGA AATAGTG	This work
ds382	ATTTAGGGAACCAGATCTTAAG	This work
ds385	CAGGACCACACTCGTCAATC	This work
**Primers used to create the *khpA*^{I61Y} amplicon**		
aw147	TACGTCTACTCTGTCCCAACTGA	This work
aw148	TCAGTTGGGACAGAGTAGACGTACGTTCTTATCGCAGA AATAGTG	This work
ds382	ATTTAGGGAACCAGATCTTAAG	This work
ds385	CAGGACCACACTCGTCAATC	This work
**Primers used to create the *khpA*^{I61C} amplicon**		
aw189	TGTGTCTACTCTGTCCCAACTGA	This work
aw190	TCAGTTGGGACAGAGTAGACACACGTTCTTATCGCAGAA A TAGTG	This work
ds382	ATTTAGGGAACCAGATCTTAAG	This work
ds385	CAGGACCACACTCGTCAATC	This work
**Primers used to create the *eloR*^{L239Y} amplicon**		
aw158	CGGTTGTAAAGATAATTTTGAGCATACAGTTGCAAGGCCT TCAAGA	This work
aw157	TATGCTCAAAATTATCTTTACAACCG	This work
ds374	CGAAACCTTGGGATACGCAG	[5]
ds377	CAGCACCCACGTTAAGCAAC	[5]
**Primers used to create the *eloR*^{L239C} amplicon**		

aw191	TGTGCTCAAAATTATCTTTACAACCGC	This work
aw192	GCGGTTGTAAAGATAATTTTGAGCACACAGTTGCAAGGCCTTCAAGA	This work
ds374	CGAAACCTTGGGATACGCAG	[5]
ds377	CAGCACCCACGTTAAGCAAC	[5]
**Primers used for fusion of 3xflag-tag to EloR**		
gs515	CCATCATGATCTTTATAATCCACTACCAGATTCCTCCTTATTTATTTC	[5]
gs516	GTGGATTATAAAGATCATGATGGTGATTATAAAGATCATGATATTGATTATAAAGATGATGATGATAAAGTGGTAGTATTTACAGGTTCAA	[5]
**Primers used for fusion of 3xflag-tag to KhpA**		
gs517	CCATCATGATCTTTATAATCCATCTGTCAACCTACTTTAAACTTATTTTG	This work
gs518	ATGGATTATAAAGATCATGATGGTGATTATAAAGATCATGATATTGATTATAAAGATGATGATGATAAAATGGATACGATTGAAAATCTCAT	This work
**Primers used to create the *khpA-sfgfp* fusion**		
ds382	ATTTAGGGAACCAGATCTTAAG	This work
aw5	TTTTTCGTCAATAACGATTCTTACTTT	This work
aw9	AAAGTAAGAATCGTTATTGACGAAAAAGGCGGCGGCGGCGGCAAACATCTTACCGGTTCTAAAGG	This work
ds233	TTATGCGGCCGCTCCACTAG	[4]
aw12	GTACAAAACTAGTGGAGCGGCCGCATAAGAAGGGCGGGACGGATG	This work
ds385	CAGGACCACACTCGTCAATC	This work
**Primers used to create the *ftsZ-mKate-Km* fusion**		
aw93	CCTGTTATTGCTCGTATCGC	This work
aw94	AGATACTTTCGTTTCCTGCCAA	This work
**Primers used to construct T18 and T25 fusions for BACTH analysis**		
mk17[a]	GAGCGGATCCCGTGGTAGTATTTACAGGTTCAAC	[5]
mk18	GCATGAATTCGAACCAGAACCACCTTCTGTATCTACAACAACATAGC	[5]
aw90	GATCTCTAGAGATGGATACGATTGAAAATCTCATTAT	This work
aw92	GATCGAATTCGATTTTTCGTCAATAACGATTCTTACTT	This work
aw113	GATCTCTAGAGGTAGTATTTACAGGTTCAACTGTT	This work
aw116	GATCGAATTCGATTCAATATCCACTTGGGCTGG	This work
aw119	GATCGAATTCGAATTGACATTGATTGTAACGTAGAAG	This work
aw120	GATCTCTAGAGCACCGTGCAGAAGTCTTGC	This work
aw114	GATCGAATTCGATTCTGTATCTACAACAACATAGCG	This work
aw121	GATCTCTAGAGGTAGCTACGGAAGTAATGGC	This work
aw122	GATCGAATTCGAATCATTGACATTGATTGTAACGTAG	This work

[a] Restriction sites are underlined.

Table S3. *E. coli* strains and plasmids used in BACTH assays.

**Name**	**Relevant characteristics**	**Reference**
XL1Blue	Host strain	Aligent technologies
BTH101	BACTH expression strain, *cya*$^-$	Euromedex
**Plasmids**		
pUT18	Plasmid used in BATCH analysis	Euromedex
pKNT25	Plasmid used in BATCH analysis	Euromedex
pUT18-khpA	T18 fused to the C-terminus of KhpA	This work
pKNT25-khpA	T25 fused to the C-terminus of KhpA	This work
pUT18-khpA^{I61F}	T18 fused to the C-terminus of KhpA ^{I61F}	This work
pKNT25-khpA^{I61F}	T25 fused to the C-terminus of KhpA ^{I61F}	This work
pUT18-khpA^{I61Y}	T18 fused to the C-terminus of KhpA ^{I61Y}	This work
pKNT25-khpA^{I61Y}	T25 fused to the C-terminus of KhpA ^{I61Y}	This work
pUT18-khpA^{R53K}	T18 fused to the C-terminus of khpA^{R53K}	This work
pKNT25-khpA^{R53K}	T25 fused to the C-terminus of khpA^{R53K}	This work
pUT18-khpA^{R59K}	T18 fused to the C-terminus of khpA^{R59K}	This work
pKNT25-khpA^{R59K}	T25 fused to the C-terminus of khpA^{R59K}	This work
pUT18-khpA^{T60Q}	T18 fused to the C-terminus of khpA^{T60Q}	This work
pKNT25-khpA^{T60Q}	T25 fused to the C-terminus of khpA^{T60Q}	This work
pUT18-khpA^{GDDG}	T18 fused to the C-terminus of khpA^{GDDG}	This work
pKNT25- khpA^{GDDG}	T25 fused to the C-terminus of khpA^{GDDG}	This work
pUT18-eloR	T25 fused to the C-terminus of EloR	[5]
pUT18-eloR$^{\Delta R3H}$	T18 fused to the C-terminus of EloR$^{\Delta R3H}$	This work
pUT18-eloR^{R3H}	T18 fused to the C-terminus of EloR^{R3H}	This work
pUT18-KH-II^{EloR}	T18 fused to the C-terminus of KH-II^{EloR}	This work
pUT18-eloR^{Jag}	T18 fused to the C-terminus of EloR^{Jag}	This work
pUT18-eloR^{L239Y}	T18 fused to the C-terminus of EloR^{L239Y}	This work

References.

1 Sievers, F. *et al.* Fast, scalable generation of high-quality protein multiple sequence alignments using Clustal Omega. *Mol Syst Biol* **7**, 539, doi:10.1038/msb.2011.75 (2011).

2 Johnsborg, O., Eldholm, V., Bjørnstad, M. L. & Håvarstein, L. S. A predatory mechanism dramatically increases the efficiency of lateral gene transfer in *Streptococcus pneumoniae* and related commensal species. *Molecular microbiology* **69**, 245-253 (2008).

3 Berg, K. H., Stamsås, G. A., Straume, D. & Håvarstein, L. S. Effects of low PBP2b levels on cell morphology and peptidoglycan composition in *Streptococcus pneumoniae* R6. *Journal of bacteriology* **195**, 4342-4354 (2013).

4 Straume, D., Stamsås, G. A., Berg, K. H., Salehian, Z. & Håvarstein, L. S. Identification of pneumococcal proteins that are functionally linked to penicillin-binding protein 2b (PBP2b). *Molecular microbiology* **103**, 99-116 (2017).

5 Stamsås, G. A. *et al.* Identification of EloR (Spr1851) as a regulator of cell elongation in *Streptococcus pneumoniae*. *Mol Microbiol* **105**, 954-967, doi:10.1111/mmi.13748 (2017).
